# To assess the knowledge, attitudes, and confidence of caregivers and administrators towards the oral health of nursing home residents in San Antonio, Texas

**DOI:** 10.1186/s12877-024-04784-x

**Published:** 2024-06-12

**Authors:** Joseph Dumbuya, Rochisha S. Marwaha, Pankil K. Shah, Suman Challa

**Affiliations:** 1 The University of Texas Health Science Center at San Antonio School of Dentistry, 7703 Floyd Curl Drive, 78229 San Antonio, TX USA; 2 The University of Texas Health Science Center at San Antonio Joe R. & Teresa Lozano Long School of Medicine, 7703 Floyd Curl Drive, 78229 San Antonio, TX USA

**Keywords:** Attitudes, Caregivers, Confidence, Knowledge, Nursing home residents, Older adults, Oral Health Care

## Abstract

**Objective:**

The primary objective of this research was to use qualitative methods to assess the knowledge, attitudes, and confidence of caregivers in their ability to provide oral hygiene assistance to residents. The secondary objective was to assess the knowledge and attitude of administrators on the provision of oral hygiene assistance for residents, and their confidence in caregivers’ ability to provide oral hygiene assistance to nursing home residents in San Antonio, Texas.

**Methods:**

A semi-structured interview guide was used to conduct face-to-face interviews with seven caregivers and twelve administrative staff from ten nursing homes in San Antonio, Texas. Employees in nursing homes who are caring for residents are referred to as caregivers and those whom they care for are referred to as nursing home residents. One survey instrument was developed for the caregiver’s knowledge, attitude, and confidence toward providing oral health care, and another to assess the administrator’s knowledge, attitude, and confidence in caregivers providing oral care for nursing home residents. The interviews were recorded, transcribed, and coded for thematic content.

**Results:**

The findings revealed that caregivers and administrators had adequate knowledge of the connection between oral and systemic health. The administrators were confident that caregivers were adequately trained to provide oral hygiene care for residents. Caregivers had a positive attitude toward the importance of good oral health. They regularly assessed the residents’ oral health, but due to time constraints, staffing shortages, and other competing tasks providing oral health care to the residents was challenging. Most caregivers were confident in their skills in providing oral care for the residents since 85.6% agreed. On the contrary, almost half of the administrators were confident that caregivers have the necessary skills to provide oral care for residents, while 41.7% were unsure.

**Conclusions:**

The study gave a broader insight into the provision of oral care in nursing home residents from the perspectives of caregivers and administrative staff. Administrators must provide caregivers with adequate training and time so they can provide adequate oral health care for the residents.

## Introduction

Since 2012, the older adult population aged 65 and older in the United States has experienced a rising trajectory and by the year 2050, this population was projected to be around 83.6 million, almost double the estimated 2012 population of 43.1 million [1]. As recent as 2011, about 4.1% of adults over the age of 65 years live in nursing homes, and about 15% of residents 85 years and older residing in nursing homes across the United States [2]. The city of San Antonio in Texas has a population of 243,000 inhabitants above the age of 60 years, and that population was expected to double by the year 2040 [3]. Older adults in nursing homes were more susceptible to poor oral health due to negligence, which led to unnecessary delays in responding to oral health issues, eventually affecting general health [4–6].

Poor oral hygiene, periodontal disease, and disease-causing bacteria in the oral cavity were associated with systemic diseases such as pneumonia, cardiovascular disease, and diabetes [6]. Pneumonia accounts for 13–48% of nursing home-related infections and had a 55% mortality rate among older adults residing in nursing homes. The aspiration of bacteria secretions from the oropharyngeal space into the lower respiratory tract was the primary pathway for bacterial pneumonia infections, particularly in patients with periodontal disease [2]. Nursing home residents with chronic diseases, degenerative nerve diseases that cause dysphagia, and those who use nasogastric or percutaneous enterogastric tubes were at a higher risk of bacteria-induced pneumonia [7]. The application of effective tooth brushing techniques, regular cleaning of dentures, and routine treatment by dentists and hygienists can decrease the rate of aspiration pneumonia in nursing home residents [6].

Most nursing home residents had poor manual dexterity and were heavily dependent on the knowledge and skills of caregivers (registered nurse (RN), licensed vocational nurse (LVN), certified nursing assistants (CNA), and medical aides) to care for their oral health. For this reason, it was essential for caregivers and administrators (social workers, Nursing home administrators, directors and assistant directors of nursing (DON) to have adequate knowledge of oral health care so they can successfully care for these residents [8]. Caregivers who were knowledgeable about caring for older adults were inclined to improve the patient’s health status, which can satisfy the expectations of the patient and their families [9]. Additionally, caregivers who had adequate knowledge, positive attitude, and skills to care for older adults in nursing homes had fewer problems in meeting the job’s daily demands and responded empathetically to the daily oral care of residents [10].

The negative attitudes of some caregivers towards oral care had affected the quality of care they provide for older adults, which can eventually lead to unfavorable oral health outcomes [11]. A cross-sectional study conducted by Lui et al. (2017) found that highly educated caregivers exhibited significant knowledge of oral health and a positive attitude toward oral hygiene care for patients [12]. Goh et al. (2016) investigated the perspectives and attitudes of caregivers toward oral care and found that caregivers had positive attitudes toward providing oral care, but about 50% lacked the confidence to provide oral care for the residents [13].

The confidence of caregivers in the delivery of oral healthcare can affect the caregiver’s ability to perform clinical tasks, quality of care, and patient’s oral health outcomes, and potentially further impact their relationships with patients and the healthcare team [14].

Studies had been performed to assess caregivers’ knowledge, attitudes, and practices regarding the oral hygiene assistance of nursing home residents internationally [12, 15, 16]. However, there were no reported studies regarding the assessment of the knowledge, attitudes, and confidence of caregivers in the provision of oral hygiene assistance to nursing home residents in the United States. This study aims to address the gaps in the literature regarding the beliefs and behaviors of caregivers and administrative staff in the provision of oral hygiene assistance for nursing home residents in San Antonio, Texas, and provide a new perspective for future studies. The primary objective of this research was to use qualitative methods to assess the knowledge, attitudes, and confidence of caregivers in their ability to provide oral hygiene assistance to residents. The secondary objective was to assess the knowledge and attitude of administrators in the provision of oral hygiene assistance for residents, and their confidence in caregivers’ ability to provide oral hygiene assistance to nursing home residents in San Antonio, Texas.

## Methods

This research was deemed exempt (protocol number: 20210714NRR) by the Institutional Review Board (IRB) at the University of Texas Health Science Center in San Antonio. Twenty nursing homes in San Antonio that were affiliated with the University of Texas Health San Antonio School of Dentistry were randomly selected and invited to participate in the study. Out of twenty nursing homes invited to participate in the study, only 10 agreed to allow their staff to participate in the study. Nursing homes in San Antonio vary in size with 20–30 caregivers and 3–5 administrators per site. The study population included caregivers and administrators working at nursing homes or long-term care facilities in San Antonio, Texas. Registered nurses (RNs) licensed vocational nurses (LVNs), certified nursing assistants (CNAs), and medical aides are referred to as caregivers while social workers, nursing home administrators, directors, and assistant directors of nursing were referred to as administrators. Out of 250 caregivers and 40 administrators from 10 nursing homes who were eligible for the study, we were only able to recruit 7 caregivers and 12 administrators using a non-probability convenience sampling method. This study was conducted during the COVID-19 pandemic, so it was difficult to get more than 19 volunteers to participate in the study.

All caregivers and administrators who had worked at the nursing homes for at least three months, were 18 years and older, communicated in the English language, and voluntarily consented to participate in the study were eligible to participate. The participants who did not meet the inclusion criteria were excluded from the study. Also, participants provided informed consent by signing a consent form when they arrived at the interview location and before engaging in the interview. The participants were not offered any incentives for their involvement in the study.

A semi-structured interview guide developed from previous oral healthcare-related studies [17–20] that examined the knowledge, attitudes, practices, and confidence of various populations regarding oral healthcare was used to conduct face-to-face interviews with caregivers and administrators at nursing homes in San Antonio. Two different survey instruments were used for interviewing the caregivers and administrators. The first part of the interviews collected demographic data for the participants, such as age, gender, experience, and education. The following sections included questions to assess the knowledge of caregivers and administrators regarding oral health and their attitudes regarding the provision of oral care for nursing home residents. The final sections focus on assessing the caregiver’s confidence and administrators’ confidence in caregivers providing daily oral care for residents.

Each interview was conducted in a private space to maintain the confidentiality of the participant and lasted for 20–30 min. The interviewer obtained written informed consent before the interview, and the interviewer asked all the questions including the demographic questions. The interviews were conducted between October 2021 and January 2022 by a member of the research team and digitally recorded on an encrypted laptop, with only the research team having access to it. Data collected from study participants were stored in a safe and locked storage space at the University. The identity and confidentiality of the participants and collected data were protected throughout the study procedures.

Interviews were digitally recorded, transcribed, and hand-coded to form themes by one team member, and a qualitative data management software technology MAXQDA was used to code and generate themes by another team member using calibration among coders. The themes were compared between the two coders and a third member of the team broke the tie if the coders disagreed on t theme. The confidence-based questions were categorized on a 5-point Likert scale as it allows for a lower margin of error and provided a deeper insight. The themes gleaned from key-informant interviews were explored among team members which were followed by a discussion for quality assurance purposes.

## Results

The demographic data of caregivers (*N* = 7) and administrative staff (*N* = 12) from 10 nursing homes across San Antonio is shown in Table 1.

**Table 1:** Study population (nursing home caregivers and administrators) characteristics

Characteristics	N(%)	(N) Caregivers	(N) Administrators
**Gender**			
Male	4(21)	1	3
Female	15(79)	6	9
**Education**			
High school diploma	4(21)	4	-
Associate degree	5(26)	3	2
College degree	2(11)	-	2
Bachelor’s degree	3(16)	-	3
Master’s degree	5(26)	-	5
**Age(years)**			
20–29	4(21)	2	2
30–39	4(21)	2	2
40–49	3(16)	1	2
50–59	6(31)	2	4
60–69	2(11)	-	2
**Experience(years)**			
1–5	3(16)	1	2
6–10	3(16)	1	3
11–15	5(26)	2	2
16–20	5(26)	3	2
21–25	2(11)	-	2
26–30	1(5)	-	1

Most of the study participants were female (79%), more than half of the caregivers had a high school education, and (43%) had an associate degree. All the administrators had an associate degree or higher and the participants’ mean work experience was 13.8 years.

Both the hand-coded and the MAXQDA software data analysis of the Interview data on the knowledge questions resulted in the themes, the connection between oral and systemic health, and training in oral health and oral healthcare. Interview data on attitudes resulted in the themes, the importance of good oral health, caregivers’ time constraints, and assessment of residents’ oral health.

### Themes related to oral health knowledge-based questions

**Theme 1: The connection between oral and systemic health**: Most caregivers and staff understood the connection between oral and systemic health. They were aware that most nursing home residents had two or more morbidities some of which may lead to mortality if not treated accordingly. Their understanding of the connection between systemic and oral diseases such as diabetes, cardiovascular disease, and the side effects of certain medications on oral health has incentivized caregivers to care for the resident’s oral health to prevent the systemic spread of disease.

“At their age, if they (resident) get a tooth infection, that can travel through their bloodstream…and the resident needs to understand that.*”* Caregiver.

“If you have poor oral hygiene, it can lead to infections in your system. You can get pneumonia; I think you can get it from poor oral health. It’s like a big problem: the chain of events that can lead to other things.” Administrator.

“The mouth is the gateway to your heart and the condition of your mouth will tell a lot of things about your health in general. If you are not getting good oral care, you are going to get more health issues as you progress in life, especially if you are elderly. “Administrator.

“If a resident has an infection from a tooth, it can travel throughout their body and may cause death…. I know in the past, we had problems with a patient because of tooth infection, which led to other issues with their health.” Administrator.

**Theme 2: Training in oral health**: Caregivers and administrators mentioned that they need more training to improve their oral health knowledge and learn new techniques to work with residents on ventilators and those with dementia who sometimes refuse or resist care. They also indicated that receiving continuing education (CE) on oral care will raise their level of awareness, increase their confidence in the delivery of oral care and hygiene, and improve health outcomes. One administrator also stressed the need to improve the curriculum of the certified nursing assistant (CNA) programs so they can get more hours of oral health education.

“More training will help a lot because sometimes we try different ways to do it, but it does not work out, so we need more information, and more training because dental care is very important.” Caregiver.

“I have been an instructor for the CNA program, and I know the training is good. If it comes from a dentist, it will probably be better regarding what the resident needs. But it is not something we can offer at this time. The training is there, but it is probably not as accepted as it should be, which needs improvement.” Administrator.

“There would be some new people coming in and may need more training on brushing teeth and doing denture care; some may be different from others. I am all up for new training and learning new things.” Caregiver.

“We need more training on the provision of oral care for residents, we need more individuals who are confident enough to train caregivers to provide oral hygiene assistance for residents. Oral care is an expertise so we need more education on that.” Administrator.

In response to the attitude-based questions, the themes that emerged were the importance of good oral health, caregivers’ time constraints, and residents’ oral health assessment.

### Themes related to oral health attitude-based questions

**Theme 3: The importance of good oral health**: Almost all participants stressed the importance of good oral health and its impact on the residents’ overall health and well-being. They acknowledged that good oral health enables residents to consume their food adequately and absorb critical micronutrients, which were essential for the growth and function of their immune cells. Administrators believed that residents with compromised immune systems were more susceptible to chronic diseases that can be fatal. Good oral health can prevent aspiration pneumonia and other chronic diseases prevalent in nursing homes.

“Good oral health contributes to the health of the body. If you have good oral health, you tend to eat more, better, and more adequately. You will also be more likely to take your medication and be more outgoing because you feel comfortable about how you look.” Caregiver.

“Residents with good oral health may have better health outcomes and be in a good mood. Good oral health makes them a whole different person, they can eat better, and their health is better.” Administrator.

“In this nursing home, good oral health was essential because many patients were under ventilator and gastrostomy. Good oral health will decrease their risk of aspiration pneumonia, which was a horrible thing sometimes, we have here.” Administrator.

“I think good oral health is important, especially for those who cannot do oral care because their disease process does not allow them to remember how to do it. It was also important for the caregivers to do it daily, and the family needs to see that oral care was performed and they are not going to visit their loved one and see food in their teeth… or a bad odor coming from their mouth.” Administrator.

**Theme 4: Caregiver’s time constraints**:. Prior to the coronavirus pandemic, some nursing homes had a proportion of 14 residents per caregiver. This situation worsened during the pandemic because nursing homes were competing with hospitals to employ caregivers. Almost all the caregivers and administrators stated that the shortage of caregivers, staff taking time off from work, absenteeism, and other competing tasks limited the time caregivers must provide oral care for the residents.

“Sometimes we have no time to care for the oral health of the residents. We have like 60 patients and 2 or 3 people to care for them, so we have no time.” Caregiver.

“I don’t think they had enough time. I had always thought you had 30 patients in a hallway, and you only had two CNAs. They usually split it, and most of them require two persons’ help, limiting their time with all the chores they must do to take care of the patients.” Administrator.

“I don’t think they are allowed enough time. If you have call-ins and you cannot get somebody to come in, then they are working short… they must be on that routine base, and they had to make sure they cover the shift and provide care for all the residents.” Administrator.

“Making sure that caregivers had enough time to do their work is a challenge. Time management is everything but it also depends on what going on with your patients, what is going on in the hall, and how you are staffed, it depends on lots of things coming into play.” Administrator.

“Our ratio of patient to caregiver is probably 1:12 and if they experience call-ins or no show then the ratio will increase from there so it is really hard to provide good oral hygiene if you are rushing from one patient to the next. Also, some of our resident’s caseloads just grow if there was a shortage of staffing and we don’t feel we had enough time as each day fluctuates. “Administrator.

**Theme 5: Assessment of residents’ oral health**: The state of Texas regulatory services for nursing homes requires that nursing home residents participate in an annual health screening to assess their oral health status so that nursing home administrators can plan and facilitate the provision of oral care for residents. Initial oral health assessment of newly admitted residents was also required for all nursing homes, served as a baseline, and was essential for planning and treating chronic oral diseases. Additionally, the regular assessment of the resident’s oral cavity helped caregivers determine the type of diet (soft or regular) that was suitable for the resident and subsequently enabled the nutritionist to plan the resident’s diet accordingly.

“As part of the initial assessment process, we examined the resident’s oral cavity to determine if they had total or partial dentures so we can plan for their oral care.” Caregiver.

“We do initial oral health assessment for the residents upon arrival to our facility…. the nurses check their oral mucosa, gums, teeth, check for oral sores, thrush, and a partial denture or edentulous so we can carefully plan for the resident’s oral care and food type.” Administrator.

“They had to assess the oral health of residents and if there were any oral issues they (caregivers) must report to the doctor, social worker, or director of nursing so they can attend to their needs.” Administrator.

“We had to assess the resident’s oral cavity for missing teeth, chipped teeth, full or partial dentures… upon admission and document it. If we fail to identify any existing oral problems upon admission and something happens later then it will be our responsibility to care for that. So, it is important that we do an initial and regular assessment of the resident’s oral health.” Administrator.

### Confidence of caregivers and administrators in the provision of oral health care

Confidence of caregivers relates to their confidence in the provision of oral care for residents. Administrators’ confidence in the provision of oral care relates to their confidence in their caregiver’s provision of oral health care.

The responses to the confidence-based questions for seven caregivers were based on a 5-point Likert scale (Table 2).

**Table 2:** Caregiver’s response to the confidence-based questions related to oral and denture care, tobacco use, and work with combative residents

Question	Strongly Agree	Agree	Unsure	Disagree	Strongly Disagree
Are you confident in……?	N (%)	N (%)	N (%)	N (%)	N (%)
providing oral care for the residents	3 (42.8)	3 (42.8)	1 (14.2)	0	0
discussing the harmful effects of tobacco use	3 (42.8)	1 (14.2)	3 (42.8)	0	0
providing denture care for the residents	3 (42.8)	2 (28.5)	2 (28.5)	0	0
dealing with combative residents	3 (42.8)	3 (42.8)	0	0	1 (14.2)

In response to questions on caregivers’ skills and denture care for residents, most caregivers agreed that they were confident in their skills to provide oral care (85.6%) and denture care (71.3%) for the residents, respectively. When asked about their confidence in providing oral care to resistive residents and discussing the harmful effects of tobacco use with the residents, most caregivers agreed that they were confident in working with combative residents (85.6%) and discussing tobacco use (57%) respectively.

The responses to the confidence-based questions for twelve administrators were also based on a 5-point Likert scale (Table 3).

**Table 3:** Administrator’s response to the confidence-based questions regarding the caregiver’s skills, time, training, care, and the resident’s diet

Question	Strongly agree	Agree	Unsure	Disagree	Strongly disagree
Are you confident……?	N (%)	N (%)	N (%)	N (%)	N (%)
in caregiver’s skills in providing oral care for residents	2(16.7)	4 (33.3)	5 (41.7)	0	1 (8.3)
that the resident’s diet has the necessary nutrients to maintain optimal oral health	6 (50)	3 (25)	3 (25)	0	0
that caregivers have adequate time to care for resident’s oral health	1 (8.3)	2 (16.7)	6 (50)	1 (8.3)	2 (16.7)
that caregivers have adequate training	0	6 (50)	4 (33.3)	1 (8.3)	1 (8.3)
that caregivers provide adequate care for the residents	3 (25)	5 (41.7)	4 (33.3)	0	0

The administrators were asked about caregivers’ confidence in providing adequate care for the residents and whether their diet contained essential nutrients for optimal oral health. Most of the administrators were confident that caregivers provided adequate care for the residents (66.7%) and that the resident’s diet contained all the nutrients to maintain optimal oral health (75%). In response to the question on caregivers’ skills to provide oral care, half of the administrators (50%were confident that their caregivers had adequate skills to provide oral care for residents, while 41.7% were unsure. Regarding training, administrators were confident that caregivers were adequately trained since 50% agreed with the statement.

## Discussion

To the best of our knowledge, this is the first study conducted in the United States that assessed the knowledge, attitudes, and confidence of caregivers and administrators in the provision of oral care for nursing home residents. Our findings showed that caregivers and administrators have adequate knowledge about the connection between oral and systemic health which is broadly consistent with previous studies [21, 22]. A 2009 study to evaluate the importance of oral health in nursing homes revealed that older adults were more susceptible to chronic systemic diseases that can affect their overall health, and periodontal disease has been linked to systemic conditions through inflammatory processes [21]. In our study, caregivers and administrators were knowledgeable about the connection between oral and systemic health and that infection in the oral cavity can metastasize to other body organs through the bloodstream. They also understood that poor oral hygiene can lead to aspiration pneumonia prevalent among nursing home residents.

Another theme that emerged from the study was the need for more training for caregivers in providing oral care for the residents, which was consistent with previous studies [23–25]. Although caregivers and administrators were knowledgeable about oral health, a vast majority of caregivers requested continuing education (CE) to improve their knowledge and enhance their skills in providing oral healthcare for hostile and resistant patients. Additionally, almost half of the administrators felt that caregivers were adequately trainedto provide oral care for residents. Administrators who were responsible for planning and implementing CE programs for caregivers stated that they could not offer such programs due to staffing shortages, lack of time, and logistical challenges involved in implementing such programs. As a result, they are highly dependent on the oral care knowledge the Certified Nursing Assistants (CNAs) acquired from their CNA training programs which may be insufficient.

Caregivers’ time constraints emerged as a significant barrier to providing care for residents, consistent with other studies (23–24,). In this study, nearly all the administrators agreed that the caregivers lack sufficient time to provide oral care for the residents due to competing tasks, and staffing shortages leading to 1 caregiver caring for 8–10 residents at a time. Additionally, it was time-consuming to provide care for residents with dementia and combative residents. In a systematic review of studies on the knowledge, attitudes, and beliefs acting as barriers and facilitators for the provision of oral care, the authors found that it takes plenty of time to provide oral care to combative patients [26]. On the contrary, in a 2011 cross-sectional study performed in Sweden, the authors found that the nursing staff believed they had sufficient time to perform oral care practices [27].

Both caregivers and administrators understood the importance of good oral health which was consistent with previous studies [20, 28]. Coleman (2002) found that effective oral care practice was about recognizing the importance and ensuring that daily oral hygiene care was given similar priority as bathing residents, combing their hair, administering medication, and other care practices [28]. In this study, caregivers believed that oral care was essential for nursing home residents, especially those on ventilators and gastrostomy. Residents with gastrostomy cannot be fed through the oral cavity resulting in neglect of oral care. Maeda and Akagi (2014) found that patients with limited oral intake or tube feeders need meticulous oral care to reduce poor clinical outcomes related to aspiration pneumonia [29].

Most participants revealed they must perform an initial assessment of the resident’s oral health before admission to their facility and whenever they complain of toothache. Some participants also explained that they sometimes could not adequately assess the resident’s teeth and periodontal structures due to a lack of cooperation by the residents. In a study performed in 2009, the authors stated that oral care was i not always adequately evaluated during assessments of the general health of residents due to lack of patient cooperation, time, restricted mouth opening, unpleasant nature of the task, lack of training, and knowledge [21].

Our study indicated that most of the caregivers agreed that they were confident in their skills in providing oral and denture care for the residents. However, administrators were confident in the caregiver’s ability and skills to provide oral and denture care for the residents. This is consistent with a 2014 study that reported that more caregivers felt confident in assisting residents with brushing their teeth than with flossing [30]. However, a cross-sectional study found that half of the caregivers lack confidence in providing oral care because of fear of harming the patient [13]. In our study, caregivers mentioned their challenges with providing oral care for residents on ventilators, but their experience had given them the confidence to provide adequate care.

One of the strengths of this study is that the interviews were conducted by a dentist who had no prior encounter with the participants, making it possible for participants to respond to the questions openly thereby reducing the potential for bias. Another strength of the study was that using qualitative methods provided insights into the attitudes of the respondents and why they agreed or disagreed with some of the questions and comments. Despite several strengths of this study, there were some limitations.

The study used a convenience sampling method to recruit participants, which may have led to selection bias. Most of the participants in the study were those who wanted to see positive change in the provision of oral care for residents, which may have accounted for response and social desirability bias. Participants may not have felt comfortable talking about their lack of confidence and abilities to a dentist for fear of being judged by an oral health expert, especially a dentist. They may have felt pressured to give good answers to look competent in the eyes of a dentist. Another limitation is that caregivers who volunteered to participate in the study may have been those who felt confident with their knowledge and skills, thus overestimating the caregiver’s knowledge and skills compared to the general caregiver population. Participant recruitment was a major challenge due to staffing shortages and COVID-19 restrictions in nursing homes. On several occasions, scheduled interviews with participants were canceled due to COVID-19 outbreaks and other emergencies which inadvertently prolonged the time for data collection. Although we assumed the sample is representative of nursing homes throughout Texas, the findings of this study may not be generalized to all nursing home residents in the United States due to possible differences in the regulation of oral care in nursing homes across states. Lastly, the question of whether caregivers had adequate time to perform oral hygiene care was not included in the survey instrument thereby limiting caregivers’ responses to the lack of adequate time to perform their daily tasks.

## Conclusion

The study gave a broader insight into the provision of oral care in nursing homes from the perspectives of caregivers and administrative staff. Administrators must provide adequate training and time to caregivers so they can provide adequate oral health care for the residents. Future research must be undertaken to investigate the role of nursing home administrators in the provision of oral care for residents.

### Public health recommendations

Implementation of a national policy for the provision of oral care for residents in long-term care settings, standardization of procedures coupled with an effective auditing system for compliance is indicated. In addition, nursing home authorities should be able to recruit and retain more caregivers through collaborative efforts with nursing training schools within the community to eliminate the problem of staffing shortages. We suggested that the oral care component of the curriculum of the CNA programs should be upgraded and enhanced in addition to being instructed by dental professionals. Lastly, nursing homes should consider appointing a dental champion who can coordinate continuing education and provision of oral hygiene and care for residents, especially those without dental insurance.

## Data Availability

The data that supports the findings of this study are available from the corresponding author upon reasonable request.
